# Autism Spectrum Disorders and Schizophrenia Spectrum Disorders: Excitation/Inhibition Imbalance and Developmental Trajectories

**DOI:** 10.3389/fpsyt.2017.00069

**Published:** 2017-05-01

**Authors:** Roberto Canitano, Mauro Pallagrosi

**Affiliations:** ^1^Division of Child Neuropsychiatry, University Hospital of Siena, Siena, Italy; ^2^Department of Psychiatry, Sapienza University of Rome, Rome, Italy

**Keywords:** autism spectrum disorders, schizophrenia spectrum disorders, psychosis, children and adolescents, excitation/inhibition imbalance

## Abstract

Autism spectrum disorders (ASD) and schizophrenia spectrum disorders (SSD) share clinical and genetic components that have long been recognized. The two disorders co-occur more frequently than would be predicted by their respective prevalence, suggesting that a complex, multifactor association is involved. However, DSM-5 maintains the distinction between ASD, with core social and communication impairments, and SSD, including schizophrenia (SCZ), with hallucinations, delusions, and thought disorder as essential features. ASD and SSD have common biological underpinnings that may emerge early in development and unfold over time. One of the hypotheses supporting the similarities in the social and cognitive disturbances of ASD and SSD relates to abnormalities in the ratio of excitatory to inhibitory cortical activity (E/I imbalance). E/I imbalance in neurodevelopmental disorders could be the consequence of abnormalities in genes coding for glutamatergic and GABAergic receptors or synaptic proteins followed by system derangements. SSD and ASD have been characterized as polygenic disorders in which to the onset and progression of disease is triggered by interactions among multiple genes. Mammalian target of rapamycin signaling is under intense investigation as a convergent altered pathway in the two spectrum disorders. Current understanding of shared and divergent patterns between ASD and SSD from molecular to clinical aspects is still incomplete and may be implemented by the research domain criteria approach.

## Autism Spectrum Disorder (ASD) and Schizophrenia Spectrum Disorder (SSD): Current Understanding

The clinical interplay and overlap between SSD and ASD have long been recognized as the two classes of disorder that share phenotypic and clinical features and a number of individuals diagnosed with ASD subsequently develop SSD symptoms (1). Currently, the relationship is further emphasized after controversies on the shared patterns and differences of the two disorders.

It has been demonstrated that the two types of disorder co-occur more frequently than would be that predicted by their respective prevalence. DSM-5 maintains a nosological distinction between ASD and schizophrenia (SCZ) in spite of overlapping in clinical characteristics. Examining the specific definition of core symptoms, there are two major criteria for ASD: “(1) persistent deficits in social communication, social interactions, social-emotional reciprocity and communicative behaviors and (2) restricted, repetitive patterns of behavior, interests or activities, including stereotyped or repetitive movements, behavioral rigidity, odd or intense interests,” and as an important additional criterion, abnormally high or low reactivity to sensory stimuli. On the other hand, the DSM-5 diagnostic criteria for SCZ specify that at least two of the following symptoms must be present: “hallucinations, delusions, disorganized speech, grossly disorganized or catatonic behavior and negative symptoms.” Furthermore, in DSM-5, the criteria have been reorganized to emphasize the variability in the severity of the psychopathology and the severity dimensions have been updated (2).

Childhood-onset schizophrenia (COS) is a subtype of SCZ defined by onset of psychotic symptoms before 13 years and the absence of any other neuropsychiatric diagnosis. Re-examination of the overlap between COS and ASD has highlighted the clinical and genetic commonalities. Remarkably, in almost half the cases of COS identified in the largest longitudinal study to date, a pervasive developmental disorder was present before the onset of psychosis (3). In contrast, and somewhat unexpectedly, prospective longitudinal studies following children with ASD into young adulthood rarely report the appearance of psychotic symptoms. SSD includes SCZ, schizofreniform disorder, schizoaffective disorder, and schizotypal personality disorder. In ASD, it is not rare to detect unusual preoccupations, unusual perceptual experiences, odd thinking, and speech. Both the shared clinical features and frequent co-occurrence point to a close relationship between SSD and ASD. To further strength this relationship, it has been reported that about 30% of children and adolescents with COS had co-morbid ASD (4). In addition, the well-known difficulty to recognize social cues from the actions of others is tightly related to deficit in theory of mind that is a characteristic feature common to both SSD and ASD (5).

In this context, it is critically important to underscore that the negative symptoms of SSD are often more disabling and more resistant to treatment than the so-called positive ones, e.g., hallucinations and delusions (6). These negative symptoms include social avoidance and emotional flatness and might be regarded as closely linked to impairments in social communication and motivation. These so-called negative symptoms of SSD might be considered to fall within the same domain of social impairment as the social difficulties characteristic of ASD. Furthermore, the disorganized or abnormal behaviors characteristic of SSD include behaviors which would meet ASD Criterion B, e.g., repeated and stereotyped movements and verbal expressions, as to DSM-5. Other pathognomonic features common to both conditions include impairments in facial recognition and emotion processing (7, 8). Patients with both ASD and SSD have been shown to have significant difficulties in interpreting social cues associated with eye gaze and deficits on theory of mind tasks—one of the hallmarks of ASD.

Autism spectrum disorders and SSD share biological underpinnings that may emerge in early neural development and unfold during subsequent childhood development (9). Abnormal neural development has been ascertained in cortical projection neurons from different brain areas including prefrontal and somatosensory regions in ASD and dorsolateral/ventrolateral prefrontal regions in SSD. It has to be mentioned that neurodevelopmental disorders are associated with known genetic abnormalities both in ASD and SSD phenotypes, as detailed in Table 1. Furthermore, epigenetic effects and alterations in copy number variants (CNVs) have been reported to contribute to abnormalities of neural circuits associated with SSD and ASD (Table 2). The risk of both disorders is increased by advanced paternal age and maternal infection/immune activation during pregnancy (10, 11). These shared patterns suggest that the two spectra are likely to represent outcomes of common pathophysiological mechanisms. The next sections describe the E/I imbalance as a candidate mechanism possibly involved.

**Table 1:** **Candidate genes validated in autism spectrum disorders (ASD) and schizophrenia spectrum disorders (SSD)**.

Gene	Function	Other phenotypes
RELN	Neuronal migration, polarization	Lissencephaly, Alzheimer’s disease
DISC1	Neural development, synaptic plasticity, mammalian target of rapamycin (mTOR) regulation	Depression, bipolar disorder
FOXP2	Regulates DISC1, CTNAP2, language, and neural development	Developmental verbal dyspraxia
BDNF	Neurotrophic factor, regulates mTOR/AKT	Alzheimer’s disease, Huntington disease
MECP2	Epigenetic regulator	Rett syndrome
UBE3A	Epigenetic regulator	Angelmann syndrome
NLGN3	Postsynaptic component coupled with NRXN	Undefined
NLG4	Postsynaptic component coupled with NXRN	ID
NRXN1	Presynaptic component coupled with NXRN	Pitt–Hopkins phenotype
SHANK3	Postsynaptic protein in glutamatergic neuron	Phelan–McDermid syndrome
CNTAP2	Cell adhesion and differentiation	ID, epilepsy, language impairment
CNTAP4		
GRIN2B	NMDA receptor subunit	ID, epilepsy
NTGN1	Axon guidance	Bipolar disorder
GABRB3	GABA receptor subunits	Bipolar disorder
GABRA5		
GAD	Conversion of glutamate to GABA	Epilepsy
CACNA1C	Voltage-dependent calcium channel subunit	Bipolar disorder, Brugada, and Timothy syndromes
SLC25A12	Mitochondrial membrane, solute channel protein	Mitochondrial disorders
OXTR/OXT	Oxytocin receptor/oxytocin gene	Undefined
ZNF804A	Transcription regulator of PRSS16, COMT	Bipolar disorders

*Modified by de Lacy and King (9)*.

*ID, intellectual disability*.

**Table 2:** **Copy number variants (CNVs) implicated in ASD and SSD**.

Region and type	Candidate genes	Phenotypes
1q21.1 Del	Unidentified	SCZ, ASD, ID, ADHD, deficit IGE
1q21.1 Dup	Unidentified	ASD, ID, ADHD
2p16.3 Del	NRXN1	SCZ, ASD, ID
3q29 Del	PAK2	ACZ, ID, ADHD
3q29 Dup	Unidentified	ID
15q11.2 Del	CYF1P1	ID, DD, SCZ, ASD, IGE, OCD, MDD
15q11-13 Dup	GABRA5, GABRB3, GABG3, and others	SCZ, ASD, ID, Ataxia
15q13.3 Del	CHRNA7	SCZ, ASD, ID
16p11.2 Del	DOC2A, ERK1	SCZ, ASD, ID, learning disorder
16p11.2 Dup	DOC2A, ERK1	SCZ, ASD, ID, DD
16p13.11 Del	NDE1	SCZ, ASD, ID
16p13.11 Dup	NDE1	SCZ, ASD, ID, ADHD, IGE
17q12	Undefined	SCZ, ASD, ID
22q11.2	PRODH, COMT, DGCR6, TRX1	SCZ, ASD, ID, epilepsy
22q11.21	PRODH, COMT, DGCR6, TRX1	ID, DD
22q13.3	SHANK3	ID, DD, ASD, SCZ

*Modified by de Lacy and King (9)*.

*ID, intellectual disability; ADHD, attention-deficit hyperactivity disorder, DD, developmental delay; OCD, obsessive–compulsive disorder; MMDD, major depressive disorder; IGE, immunoglobulin E deficit*.

## E/I Imbalance in ASD and SSD

An emerging hypothesis for the similarities in the social and cognitive disturbances associated with ASD and SSD is based on alterations in the ratio of excitatory to inhibitory cortical activity (E/I imbalance). Glutamate and GABA are, respectively, the two main neurotransmitters involved in excitatory and inhibitory signaling in the brain. Increased glutamatergic signaling alongside decreased GABAergic signaling would represent an E/I imbalance. Such imbalances may arise from disturbances in neural circuit formation or, abnormalities in the genes which code for proteins involved in these processes and linkage and association studies have been implicated in ASD and SSD (12). Postmortem studies have reported structural changes in both excitatory glutamatergic and inhibitory GABAergic circuits in individuals with ASD and SCZ (13–15).

In neurodevelopmental disorders, an E/I imbalance could arise directly through alterations in genes coding for glutamatergic receptors or synaptic proteins (16–18). The synapse organizers neurexins and their binding neuroligins are implicated in the formation and maintenance of excitatory and inhibitory synapses. Heterozygous deletions eliminating exons of the neurexin-1α gene in patients with ASD and SCZ have been detected and the functional significance of this recurrent deletion is still unclear. However, the availability of mice with deletion of the promoter and first exon of neurexin-1α provided evidence of the effects of neurexin-1α disruption on phenotypes relevant to ASD and SCZ and supported the role of neuroxins in neurodevelopmental disorders (19, 20).

In addition to the synaptic dysfunction, there is increasing evidence that E/I balance is also modulated by glial mechanisms that regulate glutamate activity (21, 22). Abnormalities in astrocyte gene expression in both ASD and SCZ have been detected (23), and reduced numbers of oligodendrocytes, impaired cell maturation, and altered gene expression of myelin/oligodendrocyte-related genes have been ascertained in SCZ (24). In turn, an increased number of activated microglia cells in adults with ASD have been found (25). As a result, glia deserves specific attention in the evaluation of E/I imbalance in these conditions.

## ASD and E/I Imbalance

The net effect of changes in glutamatergic and GABAergic systems in ASD may be an overall increase in the ratio of excitation to inhibition (E/I). Such an increase is likely to be implicated in seizures, macrocephaly, and core ASD symptoms (26). The E–I ratio in neocortical structures is determined by pyramidal glutamatergic neurons and inhibitory GABAergic parvalbumin (PV)-positive interneurons that are modulated and fine-tuned by minicolumns (groups of functionally autonomous neurons whose afferent and efferent connections influence the functioning of microcircuits) which have been found to be abnormal in ASD (27, 28). There are a number of candidate mechanisms for glutamatergic hyperactivity-driven hyperexcitability. Neuroligins (NL1–4) and neurexins (Nrxns 1–3) have been linked with ASD via point mutations and truncations, and chromosomal rearrangements have been identified in the region of interest (29–31). SHANK1, SHANK2, and SHANK3 are scaffolding proteins which influence the postsynaptic density of glutamatergic synapses and are of primary importance in ASD. SHANK3 is reported to be involved in Phelan–McDermid syndrome a form of ASD associated with moderate to severe intellectual disability (ID) and poor language skills (32). Regarding SHANK2 and SHANK1, they were found altered in ASD associated with mild ID as well as in high functioning individuals (33).

As to the mechanisms of GABAergic inhibitory dysfunction, the link with core ASD symptoms in humans is still under investigation. Deficit in binocular rivalry, a visual function that is thought to rely on the balance of excitation/inhibition in visual cortex has been observed in ASD individuals. The link between GABA and binocular rivalry dynamics was found specifically absent in ASD pointing to an insufficient GABA inhibitory function (34). Postmortem studies have provided evidence of alterations in GABAergic circuits in ASD individuals; there have been reports of significantly reduced GAD65/GAD67 levels in the parietal cortex and cerebellum (35).

Induced pluripotent stem cells (iPSCs) have been used to investigate putative abnormalities in neural substrate of individuals with ASD. Even if no known underlying genomic mutation could be identified in a new study herein presented, interestingly, transcriptome and gene network analyses revealed upregulation of genes involved in cell proliferation, neuronal differentiation, and synaptic formation. The main finding was that overexpression of the transcription factor FOXG1 was responsible for the overproduction of GABAergic neurons, shifting the E/I balance toward inhibition (36).

## SSD and E/I Imbalance

Several postmortem studies detected lower levels of PV mRNA and GAD67, the principal synthesizing enzyme for GABA, in dorso-lateral-prefrontal cortex (DLPFC) PV neurons of patients with SCZ. Markers of GABA neurotransmission between chandelier neurons and their synaptic targets are altered in the DLPFC of subjects with SCZ (37, 38).

NMDA receptors are ionotropic glutamate receptors involved in synaptic regulation of E/I balance and there are multiple subtypes of NMDA receptor with different functions and distributions (39). Dysfunction of NMDARs has been documented in SCZ both in experimental models and human studies. In the NMDA-hypofunction model of the disease, changes in E/I balance and the resulting changes in behaviors have been hypothesized (40). Disrupted NMDAR function is implicated in altered neurodevelopment and may play a role in the progression of symptoms for SCZ especially for cognitive deficits (41–43). NMDA receptor hypofunction has been proposed in ASD as well and the NR2A, NR2B, and NR2C genes abnormalities have been associated with ASD (44).

Remarkably, two *de novo* mutations in the *GRIN2A*-coded subunit of NMDA receptors have been detected in patients with SCZ and one *de novo* mutation in *GRIN2B*-coded subunit in a patient with ASD. Truncating mutations in *GRIN2C, GRIN3A*, and *GRIN3B* were identified in both patients and controls, but no truncating mutations were found in the *GRIN1, GRIN2A, GRIN2B*, and *GRIN2D* genes (45).

NRG1and ErbB4 genes deserve attention, are expressed at excitatory synapses, and regulate spine structure and function. ErbB4 deletion is associated with neurodevelopmental abnormalities that are consistent with SSD (46, 47). The disrupted in SCZ 1 gene (*DISC1*) is another important candidate gene implicated at different levels of neurodevelopment through a scaffolding protein and different mutations have been detected in SCZ emphasizing its role (48, 49).

E/I imbalance has been proposed as a mechanism for hallucinations, one of the main positive symptoms of SSD. Hallucinations have been linked to inhibitory deficits such as impaired GABA transmission unfolding in a series of abnormalities such as impaired NDMA receptor plasticity, reductions in gamma frequency oscillations, sensory cortical hyperactivity, and cognitive inhibition deficits. However, the mechanisms by which E/I dysfunctions at the cellular level might be linked to clinical symptoms and cognitive deficits remain unclear (50).

The 22q11 microdeletion syndrome is the most common CNV associated with SCZ as it is present in 1–2% of cases, further there is a very high association of the syndrome with SCZ, up to 30–40%. This elevated risk is not associated with any other neurogenetic syndrome. Social cognition is impaired in 22q11.2 deletion syndrome and remarkably this feature is correlated with psychotic symptoms. The role of this microdeletion as a potential contributor to E/I imbalance is undefined (51).

## Convergent Pathways vs. Divergent Phenotype in ASD and SSD

Schizophrenia spectrum disorders and ASD have been described as polygenic disorders in which the onset and progression of disease are triggered by interactions among multiple susceptibility genes.

Overlaps of risk genes among ASD and SSD have been documented. Two lines of mutant mice with *Shank3* mutations linked to ASD and SSD have been documented with shared and distinct synaptic and behavioral phenotypes. Mice with the ASD-linked InsG3680 mutation manifest striatal synaptic transmission defects before weaning age and impaired juvenile social interaction, coinciding with the early onset of ASD symptoms. On the other hand, adult mice carrying the SCZ-linked R1117X mutation demonstrated synaptic defects in prefrontal cortex and social dominance behavior. This is a paradigmatic example of different alleles of the same gene that have distinct phenotypes at molecular, synaptic, and circuit levels which may inform exploration of these divergences in human patients (52).

## Mammalian Target of Rapamycin (mTOR) Signaling in ASD and SSD

The mTOR pathway is directly involved in the physiological maintenance of the synaptic E/I ratio and is implicated in ASD by virtue of its role in upstream signaling and downstream regulatory mechanisms (12). Dysregulation of mTOR increases excitability and decreases inhibition thus contributing to E/I imbalance. mTOR activation is found in tuberous sclerosis complex mutations (TSC1/TSC2) occurring in tuberous sclerosis, which is frequently associated with ASD. Dysregulation of the mTOR pathway in these conditions provides clues to the molecular pathophysiology of ASD as the synaptic and cellular alterations involved may converge to produce the core social impairment of these disorders (53). In addition, mTOR inhibitor compounds have the potential to reverse many of the behavioral and neurophysiological abnormalities associated with ASD (54).

Recent investigations have linked SSD to the mTOR signaling cascade (55). Dysfunction of diverse upstream activators and environmental stressors, that have been previously implicated in SCZ, can lead to either over-activation or inhibition of the signaling pathway. Alterations in GABA signaling may be involved in the dysfunction of inhibitory circuits in SSD through the DISC1–Akt–mTOR pathway. As well, a putative depression of mTOR signaling with possible variation between and within brain regions affecting neuronal functioning in variable fashion has been proposed. Consistently, a preponderant decrease in glutamatergic activity with respect to GABAergic activity has been reported (56). In this functional and still undefined background, abnormal synaptic function may be related to positive and negative symptoms of SSD (57). Lastly, mTOR signaling undergoes variations as neurodevelopment unfold and environment plays a significant role especially through early life experiences that needs to be thoroughly considered (58).

## Final Remarks and Future Directions

There is epidemiological, clinical, neurobiological, and genetic evidence for a close relationship between ASD and SSD, and significant overlap in symptoms is frequently observed; however, there are also differences in clinical presentation, behavioral phenotype, and developmental trajectory.

The complex pathways that control E/I balance provide a framework for understanding how different genetic alterations implicated in these two distinct disorders can interact to disrupt excitatory and inhibitory neuronal function, neuronal circuit organization thus eventually influence complex social and cognitive behaviors. Nonetheless, it has to be clearly stated that current knowledge of the mechanistic relationships between E/I imbalance and the two spectrum disorders is still exploratory and need further evidence. The ways in which these shared mechanisms contribute to specific phenotypes such as ASD and SSD are still largely unknown. There are a number of open questions that need to be addressed such as whether there is a critical period for an E/I imbalance that mediates ASD- and SSD-associated behavior, or whether the E/I imbalance is circuit specific. Furthermore, an E/I imbalance may arise not only from synaptic dysfunction but also from altered cell fate that can lead to abnormal proportions of inhibitory and excitatory cells.

Shedding light on the shared functions of candidate genes for involvement in ASD and SSD is the key to translating genetic findings into descriptions of developmental and clinical subtypes. As to neuronal dysfunction hypothesized, abnormalities might be specific affecting only a subset of synapses in a selective group of neurons responsible of distinct symptoms but all that is still an hypothesis as evidence on the brain circuits potentially involved is lacking.

Research domain criteria (RDoC) project seems particularly indicated to this scope as it is directed to implement all the above level of understanding. One of the main purpose is to investigate mental illness through the dimensional approach to the fundamental components of behavior, through individual symptoms or symptom clusters, that cut across diagnoses, in this case specifically in ASD and SSD domains (59). Aim and legacy of RDoC novel approach is to build a research perspective that reflects advances in genetics, neuroscience, and behavioral science to provide a foundation for precision diagnosis and treatment of complex mental disorders such as those herein examined. The details obtained by the use of RDoC matrix likely will help to shed light on ASD and SSD relationships as well as on the longitudinal monitoring of emerging convergent and divergent symptoms of the two spectra (60).

It should also be noted that there is a subset of individuals with complex neurodevelopmental disorders whose symptoms span multiple functional domains including cognition and social communication. These individuals do not fit under any of the current diagnostic labels listed under ASD and SSD and further research through an RDoC approach holds promise to describe the specific biobehavioral profiles and thus eventually establish the diagnostic category in which they should be included. Consistent developmental designs are awaited to capture changes in the underlying neural circuitry, molecular pathways including E/I balance, and other biological components in ASD and SSD, relating them to changes in their corresponding cognitive and affective determinants as they emerge over time and alter behavior under the influencing role of environment.

## Author Contributions

RC and Mauro Pallagrosi equally participated in the substantial contribution to the conception or design of the work; drafted the work and revised it critically for important intellectual content; approved the final version to be published; and are accountable for all aspects of the work in ensuring that questions related to the accuracy or integrity of any part of the work are appropriately investigated and resolved.

## Conflict of Interest Statement

The authors declare that the research was conducted in the absence of any commercial or financial relationship that could be constructed as a potential conflict of interest.

## References

[1] Hommer RE Swedo SE Schizophrenia and autism-related disorders. Schizophr Bull (2015) 41(2):313–4. 10.1093/schbul/sbu188 25634913 PMC4332956

[2] American Psychiatric Association. Diagnostic and Statistical Manual of Mental Disorders. 5th ed Washington, DC: American Psychiatric Association (2013).

[3] Rapoport J Chavez A Greenstein D Addington A Gogtay N. Autism spectrum disorders and childhood-onset schizophrenia: clinical and biological contributions to a relation revisited. J Am Acad Child Adolesc Psychiatry (2009) 48(1):10–8. 10.1097/CHI.0b013e31818b1c63 19218893 PMC2664646

[4] Driver DI Gogtay N Rapoport J. Childhood onset schizophrenia and early onset schizophrenia spectrum disorders. Child Adolesc Psychiatr Clin N Am (2013) 22(4):539–55. 10.1016/j.chc.2013.04.001 24012072 PMC3771646

[5] King BH Lord C. Is schizophrenia on the autism spectrum? Brain Res (2011) 1380:34–41. 10.1016/j.brainres.2010.11.031 21078305

[6] Cochran DM Dvir Y Frazier JA ‘Autism-plus’ spectrum disorders: intersection with psychosis and the schizophrenia spectrum. Child Adolesc Psychiatr Clin N Am (2013) 22(4):609–27. 10.1016/j.chc.2013.04.005 24012076

[7] Marwick K Hall J Social cognition in schizophrenia: a review of face processing. Br Med Bull (2008) 88(1):3–58. 10.1093/bmb/ldn035 18812413

[8] Sasson NJ Pinkham AE Weittenhiller LP Faso DJ Simpson C. Context effects on facial affect recognition in schizophrenia and autism: behavioral and eye-tracking evidence. Schizophr Bull (2016) 42(3):675–8. 10.1093/schbul/sbv176.3 26645375 PMC4838097

[9] de Lacy N King BH. Revisiting the relationship between autism and schizophrenia: toward an integrated neurobiology. Annu Rev Clin Psychol (2013) 9:555–87. 10.1146/annurev-clinpsy-050212-185627 23537488

[10] McGrath JJ Petersen L Agerbo E Mors O Mortensen PB Pedersen CB. A comprehensive assessment of parental age and psychiatric disorders. JAMA Psychiatry (2014) 71(3):301–9. 10.1001/jamapsychiatry.2013.4081 24452535

[11] Knuesel I Chicha L Britschgi M Schobel SA Bodmer M Hellings JA Maternal immune activation and abnormal brain development across CNS disorders. Nat Rev Neurol (2014) 10(11):643–60. 10.1038/nrneurol.2014.187 25311587

[12] Gao R Penzes P. Common mechanisms of excitatory and inhibitory imbalance in schizophrenia and autism spectrum disorders. Curr Mol Med (2015) 15(2):146–67. 10.2174/1566524015666150303003028 25732149 PMC4721588

[13] Chattopadhyaya B Cristo GD. GABAergic circuit dysfunctions in neurodevelopmental disorders. Front Psychiatry (2012) 3:51. 10.3389/fpsyt.2012.00051 22666213 PMC3364508

[14] Glausier JR Lewis DA Dendritic spine pathology in schizophrenia. Neuroscience (2013) 251:90–107. 10.1016/j.neuroscience.2012.04.044 22546337 PMC3413758

[15] Hutsler JJ Zhang C. Increased dendritic spine densities on cortical projection neurons in autism spectrum disorders. Brain Res (2010) 1309:83–94. 10.1016/j.brainres.2009.09.120 19896929

[16] Silverman JL Smith DG Rizzo SJ Karras MN Turner SM Tolu SS Negative allosteric modulation of the mGluR5 receptor reduces repetitive behaviors and rescues social deficits in mouse models of autism. Sci Transl Med (2012) 4(131):131–51. 10.1126/scitranslmed.3003501 22539775 PMC4904784

[17] Silverman JL Pride MC Hayes JE Puhger KR Butler-Struben HM Baker S GABAB receptor agonist r-baclofen reverses social deficits and reduces repetitive behavior in two mouse models of autism. Neuropsychopharmacology (2015) 40(9):2228–39. 10.1038/npp.2015.66 25754761 PMC4613612

[18] Spooren W Lindemann L Ghosh A Santarelli L. Synapse dysfunction in autism: a molecular medicine approach to drug discovery in neurodevelopmental disorders. Trends Pharmacol Sci (2012) 33(12):669–84. 10.1016/j.tips.2012.09.004 23084458

[19] Reichelt AC Rodgers RJ Clapcote SJ. The role of neurexins in schizophrenia and autistic spectrum disorder. Neuropharmacology (2012) 62(3):1519–26. 10.1016/j.neuropharm.2011.01.024 21262241

[20] Grayton HM Missler M Collier DA Fernandes C Altered social behaviours in neurexin 1alpha knockout mice resemble core symptoms in neurodevelopmental disorders. PLoS One (2013) 8:e67114 10.1371/journal.pone.0067114 23840597 PMC3696036

[21] Di Benedetto B Rupprecht R. Targeting glia cells: novel perspectives for the treatment of neuropsychiatric diseases. Curr Neuropharmacol (2013) 11:171–85. 10.2174/1570159X11311020004 23997752 PMC3637671

[22] Durieux AMS Fernandes C Murphy D Labouesse MA Giovanoli S Meyer U Targeting glia with N-acetylcysteine modulates brain glutamate and behaviors relevant to neurodevelopmental disorders in C57BL/6J mice. Front Behav Neurosci (2015) 9:343. 10.3389/fnbeh.2015.00343 26696857 PMC4677305

[23] Fatemi SH Reutiman TJ Folsom TD Thuras PD. GABA(A) receptor downregulation in brains of subjects with autism. J Autism Dev Disord (2009) 39(2):223–30. 10.1007/s10803-008-0646-7 18821008 PMC2697059

[24] Bernstein HG Steiner J Guest PC Dobrowolny H Bogerts B. Glial cells as key players in schizophrenia pathology: recent insights and concepts of therapy. Schizophr Res (2015) 161(1):4–18. 10.1016/j.schres.2014.03.035 24948484

[25] Onore C Careaga M Ashwood P. The role of immune dysfunction in the pathophysiology of autism. Brain Behav Immun (2012) 26(3):383–92. 10.1016/j.bbi.2011.08.007 21906670 PMC3418145

[26] Uzunova G Pallanti S Hollander E. Excitatory/inhibitory imbalance in autism spectrum disorders: implications for interventions and therapeutics. World J Biol Psychiatry (2016) 17(3):174–86. 10.3109/15622975.2015.1085597 26469219

[27] Hutsler JJ Casanova M. Review: cortical construction in autism spectrum disorder: columns, connectivity and the subplate. Neuropathol Appl Neurobiol (2016) 42(2):115–34. 10.1111/nan.12227 25630827

[28] Opris I Casanova MF. Prefrontal cortical minicolumn: from executive control to disrupted cognitive processing. Brain (2014) 137(7):1863–75. 10.1093/brain/awt359 24531625 PMC4065017

[29] Etherton MR Tabuchi K Harma SM Ko J Sudhof TC. An autism-associated point mutation in the neuroligin cytoplasmic tail selectively impairs AMPA receptor-mediated synaptic transmission in hippocampus. EMBO J (2011) 30(14):2908–19. 10.1038/emboj.2011.182 21642956 PMC3160244

[30] Zhang C Milunsky JM Newton S Ko J Zhao G Maher TA A neuroligin-4 missense mutation associated with autism impairs neuroligin-4 folding and endoplasmic reticulum export. J Neurosci (2009) 29(35):10843–54. 10.1523/JNEUROSCI.1248-09.2009 19726642 PMC2777970

[31] Gauthier J Siddiqui TJ Huashan P Yokomaku D Hamdan FF Champagne N Truncating mutations in NRXN2 and NRXN1 in autism spectrum disorders and schizophrenia. Hum Genet (2011) 130(4):563–73. 10.1007/s00439-011-0975-z 21424692 PMC3204930

[32] Kolevzon A Angarita B Bush L Wang AT Frank Y Yang A Phelan-McDermid syndrome: a review of the literature and practice parameters for medical assessment and monitoring. J Neurodev Disord (2014) 6:39. 10.1186/1866-1955-6-39 25784960 PMC4362650

[33] Leblond CS Nava C Polge A Jauthier G Huguet G Lumbroso S Meta-analysis of SHANK mutations in autism spectrum disorders: a gradient of severity in cognitive impairments. PLoS Genet (2014) 10(9):e1004580. 10.1371/journal.pgen.1004580 25188300 PMC4154644

[34] Robertson CE Ratai EM Kanwisher N. Reduced GABAergic action in the autistic brain. Curr Biol (2016) 26(1):80–5. 10.1016/j.cub.2015.11.019 26711497

[35] Oblak AL Gibbs TT Blatt GJ. Decreased GABA(B) receptors in the cingulate cortex and fusiform gyrus in autism. J Neurochem (2010) 114(5):1414–23. 10.1111/j.1471-4159.2010.06858.x 20557420 PMC2923229

[36] Mariani J Coppola G Zhang P Abyzov A Provini L Tomasini L FOXG1-dependent dysregulation of GABA/glutamate neuron differentiation in autism spectrum disorders. Cell (2015) 16(162):375–90. 10.1016/j.cell.2015.06.034 26186191 PMC4519016

[37] Lewis DA The chandelier neuron in schizophrenia. Dev Neurobiol (2011) 1(71):118–27. 10.1002/dneu.20825 PMC3116957 21154915

[38] Gonzalez-Burgos G Lewis DA. GABA neurons and the mechanisms of network oscillations: implications for understanding cortical dysfunction in schizophrenia. Schizophr Bull (2008) 34(5):944–61. 10.1093/schbul/sbn070 18586694 PMC2518635

[39] Paoletti R Bellone C Zhou Q NMDA receptor subunit diversity: impact on receptor properties, synaptic plasticity and disease. Nat Rev Neurosci (2013) 14(6):383–400. 10.1038/nrn3504 23686171

[40] Kehrer C Maziashvili N Dugladze T Gloveli T. Altered excitatory-inhibitory balance in the NMDA-hypofunction model of schizophrenia. Front Mol Neurosci (2008) 8(1):6. 10.3389/neuro.02.006.2008 18946539 PMC2525998

[41] Balu DT. The NMDA receptor and schizophrenia: from pathophysiology to treatment. Adv Pharmacol (2016) 76:351–82. 10.1016/bs.apha.2016.01.006 27288082 PMC5518924

[42] Fromer M Pocklington AJ Kavanagh DH Williams HJ Dwyer S Gormley P De novo mutations in schizophrenia implicate synaptic networks. Nature (2014) 506(7487):179–84. 10.1038/nature12929 24463507 PMC4237002

[43] Snyder MA Gao WJ. NMDA hypofunction as a convergence point for progression and symptoms of schizophrenia. Front Cell Neurosci (2013) 27(7):31. 10.3389/fncel.2013.00031 23543703 PMC3608949

[44] Voineagau I Wang X Johnston P Tian Y Horvath S Mill J Transcriptomic analysis of autistic brain reveals convergent molecular pathology. Nature (2011) 474(7351):380–4. 10.1038/nature10110 21614001 PMC3607626

[45] Tarabeux J Kebir O Gauthier J Hamdan FF Xiong L Piton A Rare mutations in N-methyl-d-aspartate glutamate receptors in autism spectrum disorders and schizophrenia. Transl Psychiatry (2011) 15(1):e55. 10.1038/tp.2011.52 22833210 PMC3309470

[46] Banerjee A Macdonald ML Borgmann-Winter KE Hahn CG. Neuregulin 1-erbB4 pathway in schizophrenia: from genes to an interactome. Brain Res Bull (2010) 83(3–4):132–9. 10.1016/j.brainresbull.2010.04.011 20433909 PMC5050041

[47] Perez-Garcia CG. ErbB4 in laminated brain structures: a neurodevelopmental approach to schizophrenia. Front Cell Neurosci (2015) 18(9):472. 10.3389/fncel.2015.00472 26733804 PMC4683445

[48] Mackie S Millar JK Porteous DJ Role of DISC1 in neural development and *schizophrenia*. Curr Opin Neurobiol (2007) 17(1):95–102. 10.1016/j.conb.2007.01.007 17258902

[49] Bradshaw NJ DJ Porteous DJ. DISC1-binding proteins in neural development, signalling and schizophrenia. Neuropharmacology (2012) 62(3):1230–41. 10.1016/j.neuropharm.2010.12.027 21195721 PMC3275753

[50] Jardri R Hugdahl K Hughes M Brunelin J Waters F Alderson-Day B Are hallucinations due to an imbalance between excitatory and inhibitory influences on the brain? Schizophr Bull (2016) 42(5):1124–34. 10.1093/schbul/sbw075 27261492 PMC4988749

[51] Schneider M Debbane M Bassett AS Chow EW Fung WL van den Bree M Psychiatric disorders from childhood to adulthood in 22q11.2 deletion syndrome: results from the International Consortium on Brain and Behavior in 22q11.2 Deletion Syndrome. Am J Psychiatry (2014) 171(6):627–39. 10.1176/appi.ajp.2013.13070864 24577245 PMC4285461

[52] Zhou Y Kaiser T Monteiro P Zhang X Van der Goes MS Wang D Mice with Shank3 mutations associated with ASD and schizophrenia display both shared and distinct defects. Neuron (2016) 89(1):147–62. 10.1016/j.neuron.2015.11.023 26687841 PMC4754122

[53] Sato A. mTOR, a potential target to treat autism spectrum disorder. CNS Neurol Disord Drug Targets (2016) 15(5):533–43. 10.2174/1871527315666160413120638 27071790 PMC5070418

[54] Wang H Doering LC Reversing autism by targeting downstream mTOR signaling. Front Cell Neurosci (2013) 7:28 10.3389/fncel.2013.00028 23533374 PMC3607786

[55] Gururajan A van den Buuse M. Is the mTOR-signalling cascade disrupted in schizophrenia? J Neurochem (2014) 129(3):377–87. 10.1111/jnc.12622 24266366

[56] Weston MC Chen H Swann JW. Multiple roles for mammalian target of rapamycin signaling in both glutamatergic and GABAergic synaptic transmission. J Neurosci (2012) 32(33):11441–52. 10.1523/JNEUROSCI.1283-12.2012 22895726 PMC3432956

[57] Fatemi SH Folsom TD. The neurodevelopmental hypothesis of schizophrenia, revisited. Schizophr Bull (2009) 35(3):528–48. 10.1093/schbul/sbn187 19223657 PMC2669580

[58] Costa-Mattioli M Monteggia LM. mTOR complexes in neurodevelopmental and neuropsychiatric disorders. Nat Neurosci (2013) 16(11):1537–43. 10.1038/nn.3546 24165680

[59] Garvey M Avenevoli S Anderson K. The National Institute of Mental Health research domain criteria and clinical research in child and adolescent psychiatry. J Am Acad Child Adolesc Psychiatry (2016) 55(2):93–8. 10.1016/j.jaac.2015.11.002 26802775 PMC4724376

[60] Cuthbert BN The RDoC framework: facilitating transition from ICD/DSM to dimensional approaches that integrate neuroscience and psychopathology. World Psychiatry (2014) 13(1):28–35. 10.1002/wps.20087 24497240 PMC3918011

